# Fat mass and fat distribution are associated with low back pain intensity and disability: results from a cohort study

**DOI:** 10.1186/s13075-017-1242-z

**Published:** 2017-02-10

**Authors:** Sultana Monira Hussain, Donna M. Urquhart, Yuanyuan Wang, Jonathan E. Shaw, Dianna J. Magliano, Anita E. Wluka, Flavia M. Cicuttini

**Affiliations:** 1 Department of Epidemiology and Preventive Medicine, School of Public Health and Preventive Medicine, Monash University, Melbourne, VIC 3004 Australia; 2 Baker IDI Heart and Diabetes Institute, Melbourne, VIC 3004 Australia

**Keywords:** Obesity, Body composition, Fat mass, Fat-free mass, Low back pain

## Abstract

**Background:**

Determining the association between body composition and low back pain (LBP) will improve our understanding of the mechanisms by which obesity affects LBP, and inform novel approaches to managing LBP. The aim of this study was t﻿o examine the relationship between body composition and LBP intensity and disability.

**Methods:**

A total of 5058 participants (44% men) of the Australian Diabetes, Obesity and Lifestyle Study were assessed for LBP intensity and disability using the Chronic Pain Grade Questionnaire (2013–2014). Body mass index (BMI) and waist circumference were directly obtained. Fat mass and percentage fat were estimated from bioelectrical impedance analysis at study inception (1999–2000).

**Results:**

Eighty-two percent of participants reported LBP, of whom 27% also reported LBP disability. BMI, waist circumference, percent fat, and fat mass were each positively associated with LBP intensity and disability at 12 years after adjustment for potential confounders. LBP intensity and disability showed significant dose-responses to sex-specific quartiles of BMI, waist circumference, percent fat and fat mass. For example, the adjusted OR for LBP intensity in women increased with increasing fat mass quartiles [Q1: 1, Q2: 1.05 (95%CI 0.84–1.32); Q3: 1.25 (1.00–1.57); and Q4: 1.78 (1.42–2.24); *p* < 0.001].

**Conclusions:**

Fat mass and distribution are associated with LBP intensity and disability, suggesting systemic metabolic factors associated with adiposity play a major role in the pathogenesis of LBP. Clarifying the mechanisms will facilitate developing novel preventive and therapeutic approaches for LBP.

**Electronic supplementary material:**

The online version of this article (doi:10.1186/s13075-017-1242-z) contains supplementary material, which is available to authorized users.

## Background

Low back pain (LBP) contributed the highest years lived with disability among a total of 291 conditions investigated in the Global Burden of Disease 2010 study, resulting in 83 million years lived with disability [1]. One in ten people suffer from LBP worldwide at any point in time [1, 2] and 70–85% of people have a LBP episode at some time in their life [2]. There has been an increase in disability, chronicity and work absenteeism attributable to this condition [3], which has had an enormous negative economic impact on individuals, families, communities, industries and governments [2, 4]. As such, understanding the aetiology and risk factors for LBP is important in reducing the significant burden accountable to this condition.

The prevalence of overweight and obesity that has contributed to 3.4 million deaths in 2010 is escalating in many countries and has been labelled as a global pandemic [5]. Overweight and obesity are associated with several musculoskeletal diseases including LBP. A systematic review [6] and afterwards a meta-analysis [7] of data from small cross-sectional and prospective cohort studies showed that both overweight and obesity increased the risk of LBP. These findings were supported by the large population-based Nord-Trøndelag Health (HUNT) Study that included over 25,000 participants [8]. Most of the studies included in the meta-analysis used body mass index (BMI) cutoff points to define overweight and obesity, while some studies used body weight and a few studies used waist circumference in combination with waist to hip ratio [7]. However, no studies in the systematic review or the meta-analysis examined fat mass or distribution.

Although obesity is a risk factor for LBP in adults, the mechanism is unclear. Most research has used weight and BMI as a measure of obesity. However, these measures do not take into account body composition i.e. fat and muscle mass. There is increasing evidence for a differential effect of fat and muscle on the risk of musculoskeletal diseases [9, 10]. For instance, there are negative effects of excess adiposity on movement patterns and on body structure [11, 12] that contribute to different disabilities including LBP. Two previous cross-sectional studies examining the association between body composition and LBP have suggested an effect of fat mass on LBP. One study of 135 participants (83.1% women) found that greater fat mass, but not lean tissue mass, was associated with high levels of LBP intensity and disability [13]. A Spanish study of 1128 twin women, with 38% unavailable data on fat mass, reported a weak association of fat percent and fat mass with LBP which was confounded by genetic and early shared environmental factors [14]. Neither of these studies had longitudinal data. Similarly, both the studies included women only [14] or women mostly [13]. However, men and women differ substantially in regard to body composition, especially adipose tissue distribution [15, 16]. Determining the gender-specific effect of body composition on LBP has the potential to improve our understanding of the mechanisms by which obesity affects LBP in men and women, and thus has the potential to provide novel approaches to managing this condition. Therefore the aim of this study was to examine the relationship between gender-specific body composition and LBP intensity and disability longitudinally in a national, population-based cohort of men and women.

## Methods

### Study participants

**Fig. 1:**
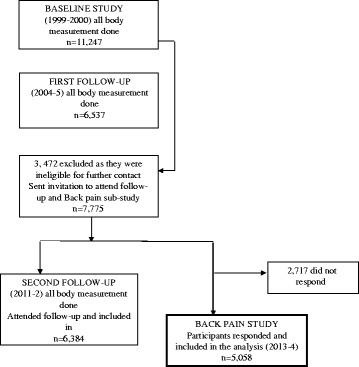
Flow diagram of recruited participants

The Australian Diabetes, Obesity and Lifestyle (AusDiab) Study is a national, population-based cohort study of 11,247 people, aged ≥25 years, recruited by a stratified cluster sampling method, involving seven strata (six states and the Northern Territory) and clusters based on census collector districts, during 1999–2000 [17]. AusDiab participants were followed up during 2004–2005 and then again in 2011–2012. Of the 11,247 participants, 3472 were excluded as they were ineligible for further contact (requested no further contact, deceased, too ill or living in high care nursing facility). In the back pain sub-study, 7775 participants were sent the back pain questionnaire between February 2013 and October 2014, of whom 5058 responded (response rate 65.1%, Fig. 1) and were included in the study. The participants who responded to the LBP questionnaire were younger, more educated, had a higher Index of Relative Disadvantage code from the Socio-Economic Indexes for Areas (SEIFA), lower BMI and waist circumference compared to those who did not respond. There was no difference in relation to body composition measures among those who responded to the LBP questionnaire and those who did not (Additional file 1: Table S1).

### Demographic, lifestyle factors, and socio-economic position assessments

Demographic and lifestyle data, including date of birth, gender, education, smoking (current, ex- or never), and leisure time physical activity (minutes per week), were collected in 1999–2000 by trained interviewers using standardised questionnaires as reported previously [17]. The Short Form 36 (SF-36), a self-administered questionnaire which measures mental and emotional conditions of a person [18, 19], was used to determine the physical (PCS) and mental health component summary (MCS) scores, and bodily pain. Socio-economic indexes for areas were estimated using the SEIFA [20]. The index is constructed such that high values reflect areas with high socio-economic position (relative advantage) and low values reflect areas with low socio-economic position (relative disadvantage).

### Obesity and body composition measurements

Data regarding obesity and body composition were measured at baseline during 1999–2000, first follow-up during 2004–2005 and second follow-up during 2011–2012. Height was measured to the nearest 0.5 cm without shoes using a stadiometer. Weight was measured without shoes and in light clothing to the nearest 0.1 kg using a mechanical beam balance. BMI was calculated as weight in kilograms divided by height in meters squared [17]. Waist circumference was measured to the nearest 1 mm using a metal anthropometric tape [21]. Fat mass was assessed by bio-impedance, using the Tanita body fat analyser (Model TBF-105, Tanita Corporation, Tokyo, Japan), which calculates body fat as a function of sex, height, weight and impedance [17, 22]. Fat percentage was calculated as fat mass divided by weight.

### Categorisation of back pain intensity and disability

The self-administered Chronic Pain Grade Questionnaire (CPGQ) was used to obtain information on LBP intensity and disability over the past 6 months (Additional file 2). This is a reliable and valid instrument for use in population surveys of LBP [13, 23, 24]. The questionnaire includes seven questions from which a pain intensity score (0–100) and disability points score (0–6) were calculated. To examine the relationship between pain intensity and various participant characteristics, subjects were classified into three groups based on their pain intensity score: no pain (=0), low pain intensity (<50), and high pain intensity (≥50). Similarly, to investigate risk factors for disability, subjects were categorized into three groups on the basis of their disability points score: no disability (=0), low disability (<3), and high disability (≥3) as previously described [13, 23, 24].

### Statistical analysis

Independent samples *t* tests for continuous variables or chi-squared tests for categorical variables were used to compare the characteristics of participants with and without LBP (LBP intensity and disability). The persistence of obesity and body composition measures were measured between baseline and first follow-up during 2004–2005. Multinomial logistic regression models were used to estimate the odds ratio (OR) with 95% confidence level (CI) for each level of LBP intensity and disability associated with each obesity and body composition measure as continuous variables. Each obesity and body composition measure was further categorized into quartiles according to their sex-specific baseline distribution. Their associations with LBP intensity and disability were analysed using ordinal logistic regression, with the lowest quartile used as the referent category. All the analysis is adjusted for age, education, smoking status, SEIFA (model 1) and further adjusted for the MCS of SF-36 in model 2. There was an additional adjustment for fat-free mass in the models where waist circumference was the explanatory variable. Furthermore, fat mass and fat-free mass were adjusted for each other. Owing to the importance of physical activity in body composition, physical activity was further adjusted in additional models with other variables. Similarly, to overcome the change in obesity and body composition measures over time further adjustment were made (n = 3032). Analysis were repeated on participants who did not have bodily pain at baseline (n = 3961). All statistical analyses were performed using Stata 14.0 (StataCorp LP., College Station, TX, USA).

## Results

The characteristics of the participants are presented in Table 1. The prevalence of low-intensity LBP was 62% (n = 3085) and high-intensity LBP was 20% (n = 1001). Most of the participants had no LBP disability 73% (n = 3061), while 16% (n = 651) reported low disability and 11% (n = 482) high disability. Participants with high LBP intensity or disability were likely to be older, less educated, current smokers, from lower SEIFA and scored lower in the SF-36 MCS component compared to those who had no LBP intensity and disability. All obesity and body composition measurements were greater in those with LBP intensity or disability compared with those without LBP symptoms. All the obesity measures (BMI, waist circumference) and body composition measures (percent fat, fat mass and muscle mass) are highly correlated at baseline and at first follow-up (correlation coefficient >0.89, *p* = <0.001) (Additional file 1: Table S2).

**Table 1:** General characteristics of participants

Low back pain intensity^a^					
	No intensity n = 900	Low intensity n = 3085	*P* No vs low	High intensity n = 1001	*P* No vs high
Age at baseline, years	48.8 (11.1)	47.3 (10.9)	0.001	51.1 (11.8)	<0.001
Female, n (%)	497 (55.2)	1682 (54.6)	0.75	598 (60.1)	0.03
Current smoker, n (%)	89 (10.1)	357 (11.2)	0.26	182 (18.6)	<0.001
University degree, n (%)	354 (39.6)	1175 (38.4)	0.54	226 (22.9)	<0.001
SEIFA (in lowest tertile), n (%)	258 (29.2)	918 (30.3)	0.86	397 (40.4)	<0.001
MCS (SF-36)	51.0 (8.5)	48.9 (9.6)	<0.001	46.3 (11.2)	<0.001
BMI, kg/m^2^	26.0 (4.7)	26.6 (4.8)	<0.001	27.8 (5.3)	<0.001
Waist circumference, cm	87.9 (13.7)	89.5 (13.7)	0.002	92.7 (14.3)	<0.001
Percent of fat (%)	31.7 (11.8)	32.6 (11.6)	0.03	36.2 (13.3)	<0.001
Fat mass, kg	24.3 (12.4)	25.7 (12.4)	0.004	29.1 (14.6)	<0.001
Fat-free mass, kg	50.6 (11.8)	51.4 (12.2)	0.10	49.3 (12.6)	0.03
Low back pain disability^b^					
	No disability n = 3,061	Low disability n = 651	*P* No vs low	High disability n = 482	*P* No vs high
Age at baseline, years	46.5 (10.7)	47.7 (11.5)	0.01	51.4 (10.9)	<0.001
Female, n (%)	1650 (54.0)	352 (54.2)	0.94	61.6 (61.2)	0.002
Current smoker, n (%)	335 (11.1)	97 (15.6)	0.01	108 (23.1)	<0.001
University degree, n (%)	1244 (41.0)	233 (36.1)	0.02	98 (20.6)	<0.001
SEIFA (in lowest tertile), (%)	888 (29.5)	212 (33.0)	0.20	194 (41.0)	<0.001
MCS (SF-36)	49.3 (9.2)	47.2 (10.6)	<0.001	46.2 (11.7)	<0.001
BMI, kg/m^2^	26.3 (4.7)	27.2 (5.0)	<0.001	28.6 (5.8)	<0.001
Waist circumference, cm	88.7 (13.5)	91.2 (13.4)	<0.001	94.1 (14.9)	<0.001
Percent of fat (%)	32.1 (11.5)	33.7 (12.6)	0.001	37.4 (13.3)	<0.001
Fat mass, kg	25.0 (12.5)	27.2 (14.0)	<0.001	30.6 (14.9)	<0.001
Fat-free mass, kg	51.4 (12.3)	51.4 (12.3)	0.95	49.1 (12.5)	<0.001

*SEIFA* Socio-Economic Indexes for Areas, *MCS* mental component score, *BMI* body mass index

^a^Data were missing for 72 participants

^b^Data were missing for 864 participants

Table 2 shows the association of LBP intensity with all anthropometric measures in men and women. All the obesity measures including BMI, waist circumference and body composition measures (percent fat, fat mass) were positively associated with LBP intensity and disability in both men and women after adjustment for age, education, smoking status, SEIFA and MCS of SF-36 (fat mass and fat-free mass were adjusted for each other). The associations between adiposity measures and high-intensity LBP were stronger than with lower-intensity LBP. Fat-free mass was negatively associated with LBP intensity only in women (low-intensity LBP: OR 0.72, 95% CI 0.54–0.96; high-intensity LBP: OR 0.75, 95% CI 0.56–1.01; *p* for between group variability <0.001 (low vs high intensity). The association did not change after introducing physical activity to the model (data not shown).

**Table 2:** Relationship between obesity and body composition and intensity of low back pain

	Male				Female			
	Number	Low-intensity pain	High-intensity pain	*P* between group	Number	Low-intensity pain	High-intensity pain	*P* between group
		OR (95% CI)	OR (95% CI)	variability (low/high)		OR (95% CI)	OR (95% CI)	variability (low/high)
Model 1								
Obesity measures								
BMI (per 5 kg/m^2^)	1	1.24 (1.07–1.44)	1.42 (1.18–1.71)	0.08	1	1.17 (1.05–1.30)	1.43 (1.26–1.62)	<0.001
Waist circumference (per 10 cm)^a^	1	1.11 (0.98–1.26)	1.26 (1.08–1.47)	0.02	1	1.14 (1.04–1.25)	1.38 (1.24–1.54)	<0.001
Body composition measures								
Percent fat (per 10%)	1	1.27 (1.08–1.49)	1.45 (1.20–1.76)	0.06	1	1.11 (1.00–1.23)	1.41 (1.25–1.59)	<0.001
Fat mass (per 10 kg)^a^	1	1.10 (0.96–1.26)	1.22 (1.04–1.42)	0.01	1	1.10 (1.01–1.19)	1.28 (1.16–1.41)	<0.001
Fat-free mass (per 10 kg)^a^	1	1.20 (0.98–1.48)	1.20 (0.93–1.56)	0.69	1	1.18 (0.94–1.48)	0.72 (0.54–0.96)	<0.001
Model 2								
Obesity measures								
BMI (per 5 kg/m^2^)	1	1.25 (1.07–1.45)	1.41 (1.17–1.70)	0.01	1	1.17 (1.05–1.30)	1.39 (1.22–1.59)	<0.001
Waist circumference (per 10 cm)^a^	1	1.11 (0.98–1.27)	1.25 (1.07–1.46)	0.06	1	1.13 (1.03–1.24)	1.36 (1.22–1.52)	<0.001
Body composition measures								
Percent fat (per 10%)	1	1.28 (1.09–1.51)	1.45 (1.19–1.77)	0.01	1	1.41 (1.25–1.59)	1.39 (1.22–1.57)	<0.001
Fat mass (per 10 kg)^b^	1	1.11 (0.97–1.27)	1.23 (1.05–1.44)	0.18	1	1.28 (1.16–1.41)	1.27 (1.15–1.40)	<0.001
Fat-free mass (per 10 kg)^b^	1	1.19 (0.97–1.47)	1.15 (0.88–1.50)	0.77	1	0.72 (0.54–0.96)	0.75 (0.56–1.01)	<0.001

Model 1: adjusted for age, education, smoking status, Socio-Economic Indexes for Areas (SEIFA), model 2: all the variables in model 1 and mental component score of SF-36

*OR* odds ratio, *CI* confidence level, *BMI* body mass index

^a^Adjusted for fat-free mass

^b^Co-adjusted for each other

The association of LBP disability with all obesity and body composition measures is shown in Table 3. Similar negative associations for all the obesity measures and body composition measures except fat-free mass were observed for LBP disability in both men and women after adjustment for age, education, smoking status, SEIFA and MCS of SF-36 (fat mass and fat-free mass were adjusted for each other). The association did not change after introducing physical activity or change in obesity and body composition measures to the model (data not shown).

**Table 3:** Relationship between obesity and body composition and low back disability

	Male				Female			
	Number	Low disability OR (95% CI)	High disability OR (95% CI)	*P* between -group variability (low/high)	Number	Low disability OR (95% CI)	High disability OR (95% CI)	*P* between group variability (low/high)
Model 1								
Obesity measures								
BMI (per 5 kg/m^2^)	1	1.04 (0.88–1.22)	1.44 (1.20–1.74)	0.002	1	1.25 (1.13–1.39)	1.49 (1.33–1.66)	0.01
Waist circumference (per 10 cm)^a^	1	1.15 (1.00–1.32)	1.36 (1.17–1.61)	0.05	1	1.23 (1.12–1.35)	1.39 (1.27–1.54)	0.03
Body composition measures								
Percent fat (per 10%)	1	1.12 (0.94–1.34)	1.42 (1.14–1.77)	0.05	1	1.21 (1.08–1.35)	1.52 (1.35–1.72)	0.002
Fat mass (per 10 kg)^b^	1	1.08 (0.94–1.24)	1.30 (1.11–1.52)	0.03	1	1.18 (1.09–1.28)	1.34 (1.23–1.47)	0.02
Fat-free mass (per 10 kg)^b^	1	0.92 (0.73–1.17)	0.86 (0.65–1.14)	0.66	1	1.05 (0.82–1.33)	0.89 (0.69–1.16)	0.28
Model 2								
Obesity measures								
BMI (per 5 kg/m^2^)	1	1.02 (0.86–1.21)	1.39 (1.15–1.68)	0.004	1	1.25 (1.12–1.39)	1.45 (1.29–1.62)	0.02
Waist circumference (per 10 cm)^a^	1	1.13 (0.98–1.30)	1.32 (1.12–1.56)	0.08	1	1.23 (1.11–1.35)	1.37 (1.23–1.52)	0.04
Body composition measures								
Percent fat (per 10%)	1	1.11 (0.92–1.32)	1.37 (1.10–1.72)	0.07	1	1.20 (1.07–1.35)	1.48 (1.31–1.68)	0.004
Fat mass (per 10 kg)^b^	1	1.07 (0.93–1.23)	1.28 (1.09–1.51)	0.04	1	1.18 (1.09–1.28)	1.32 (1.21–1.45)	0.04
Fat-free mass (per 10 kg)^b^	1	0.93 (0.73–1.18)	0.85 (0.63–1.13)	0.61	1	1.09 (0.85–1.39)	0.93 (0.72–1.22)	0.29

Model 1: adjusted for age, education, smoking status, Socio-Economic Indexes for Areas (SEIFA), model 2: all the variables in model 1 and mental component score of SF-36

*OR* odds ratio, *CI* confidence level, *BMI* body mass index,

^a^Adjusted for fat-free mass

^b^Co-adjusted for each other

BMI, waist circumference, percent fat and fat mass were significantly and positively related to LBP intensity and disability for both men and women for sex-specific quartile cutoff points of these measures after adjustment for confounders (Table 4, *p* <0.04). For example, the adjusted OR for women for fat mass in relation to LBP intensity increased from quartile 1 to quartile 4 (Q1: reference category, Q2: OR 1.05, 95% CI 0.84–1.32; Q3: OR 1.25, 95% CI 1.00–1.57; and Q4: OR 1.78, 95% CI 1.42–2.24; *p* <0.001). A similar trend was observed for LBP disability (Q1: reference category, Q2: OR 1.07, 95% CI 0.79–1.45; Q3: OR 1.37, 95% CI 1.03–1.83; and Q4: OR 2.30, 95% CI 1.74–3.04; *p* <0.001). In contrast, fat-free mass was negatively associated with LBP intensity in women. No such association was observed for either LBP disability in women, or for LBP intensity or LBP disability in men.

**Table 4:** Low back pain intensity and disability in relation to measures of sex-specific quartiles

	Q1	Q2	Q3	Q4	*P* for trend
Low back pain intensity, OR (95% CI)					
Men					
Obesity measures					
Body mass index	1	1.47 (1.14–1.88)	1.75 (1.36–2.25)	1.50 (1.16–1.93)	0.001
Waist circumference^a^	1	1.43 (1.10–1.84)	1.78 (1.36–2.34)	1.50 (1.12–2.00)	0.003
Body composition measures					
Percent fat	1	1.58 (1.23–2.03)	1.64 (1.28–2.12)	1.61 (1.25–2.08)	<0.001
Fat mass^b^	1	1.78 (1.38–2.30)	1.91 (1.46–2.51)	1.51 (1.14–1.99)	0.02
Fat-free mass^b^	1	0.88 (0.68–1.14)	0.89 (0.68–1.16)	1.00 (0.76–1.33)	0.43
Women					
Obesity measures					
Body mass index	1	1.25 (1.01–1.56)	1.25 (1.00–1.56)	1.92 (1.53–2.41)	<0.001
Waist circumference^a^	1	1.20 (0.96–1.50)	1.42 (1.13–1.78)	2.09 (1.65–2.65)	<0.001
Body composition measures					
Percent fat	1	1.01 (0.81–1.26)	1.21 (0.96–1.51)	1.79 (1.42–2.24)	<0.001
Fat mass^b^	1	1.05 (0.84–1.32)	1.25 (1.00–1.57)	1.78 (1.42–2.24)	<0.001
Fat-free mass^b^	1	0.85 (0.68–1.06)	0.94 (0.75–1.17)	0.79 (0.63–0.99)	0.01
Low back pain disability, OR (95% CI)					
Men					
Obesity measures					
Body mass index	1	1.12 (0.82–1.53)	1.13 (0.83–1.54)	1.31 (0.96–1.79)	0.001
Waist circumference^a^	1	1.59 (1.15–2.21)	1.29 (0.91–1.83)	1.85 (1.28–2.66)	0.003
Body composition measures					
Percentage fat	1	1.12 (0.82–1.54)	1.20 (0.87–1.64)	1.37 (1.00–1.88)	0.04
Fat mass^b^	1	1.20 (0.87–1.66)	1.26 (0.90–1.76)	1.45 (1.02–2.05)	0.04
Fat-free mass^b^	1	1.00 (0.73–1.37)	0.75 (0.54–1.05)	0.78 (0.55–1.12)	0.07
Women					
Obesity measures					
Body mass index	1	1.36 (1.01–1.83)	1.58 (1.18–2.12)	2.56 (1.93–3.40)	<0.001
Waist circumference^a^	1	1.16 (0.86–1.58)	1.53 (1.15–2.06)	2.50 (1.86–3.34)	<0.001
Body composition measures					
Percent fat	1	1.08 (0.80–1.45)	1.49 (1.12–1.99)	2.15 (1.63–2.85)	<0.001
Fat mass^b^	1	1.07 (0.79–1.45)	1.37 (1.03–1.83)	2.30 (1.74–3.04)	<0.001
Fat-free mass^b^	1	1.20 (0.91–1.58)	1.16 (0.88–1.54)	.92 (0.69–1.22)	0.86

Adjusted for age, sex, education, smoking status, Socio-Economic Indexes for Areas (SEIFA) and mental component score of SF-36

*OR* odds ratio, *CI* confidence level

^a^Adjusted for fat-free mass

^b^Co-adjusted for each other

Repeating the analysis in Tables 2, 3 and 4 including only those with no bodily pain at baseline (n = 3961) yielded similar results (data not shown).

## Discussion

The results from this large, prospective, population-based cohort study showed that obesity measures (BMI and waist circumference), percent fat, and fat mass were positively associated with LBP intensity and disability, independent of fat-free mass. With increasing sex-specific quartiles of obesity measures, fat percent and fat mass, the risk of LBP intensity and disability increased in a linear manner. Fat-free mass was negatively associated with LBP intensity in women only, with LBP intensity reduced in a linear manner in relation to increasing sex-specific quartiles of fat-free mass, independent of the potential confounders and fat mass.

The results of the current study, that LBP is associated with overweight and obesity measures, are consistent with a recently published systematic review [6] and a meta-analysis [7] of mostly cross-sectional studies and the results from the large HUNT cross-sectional study [8]. The present study extends these findings by providing evidence from a longitudinal study, thus strengthening the evidence for this relationship. In addition we also demonstrated that obesity measures are associated with both LBP intensity and disability and that the strength of association increases with the severity of LBP, so a dose–response relationship.

The current study demonstrated a longitudinal relationship between fat mass and percent fat and LBP. The only previous data were from two cross-sectional studies [13, 14], including one that was a twin study [14]. Furthermore, this study showed a dose–response relationship of increased LBP intensity and disability in relation to increasing quartile of fat percent and fat mass measures. In contrast, the effect of fat-free mass was less consistent, with some evidence for reduced fat-free mass as a risk factor of LBP intensity in women. While most previous studies examining the association between muscle mass and LBP did not report any significant findings [25–27], only one study reported that reduced trunk and lower extremity muscle mass were associated with chronic LBP measured by a negative straight leg raise test, in a subgroup of 71 women [28]. The straight leg raise test has been reported to have limited diagnostic accuracy [29]. Moreover, the study did not adjust for potential confounders, particularly the independent effect of muscle and fat mass, even though both are strongly associated with BMI and individually may simply reflect obesity. Though there is a paucity of evidence, it is possible that fat-free mass may have a beneficial effect on reducing LBP through increasing back muscle strength and back muscle endurance, which ultimately improve spinal health [30]. However, this needs further exploration.

By imposing continuous high biomechanical loading on the intervertebral disc of the lower back, obesity may result in structural modification of the disc [31, 32] that leads to greater low back pain and/or disability. Increased muscle mass having an inconsistent effect on LBP intensity and disability, and increased fat mass associated with LBP intensity and disability both in men and women suggest that the effect of obesity in the pathogenesis of LBP is mainly mediated through fat mass, despite differences in degrees of obesity. The mechanism of involvement of fat mass in the pathogenesis of LBP is likely to be multifactorial: it occurs via excess fat placing increased load on the spine; alternatively, the effect of fat mass may occur through systemic processes. There is evidence that fat mass is metabolically active and may adversely affect structures through systemic inflammatory processes induced by pro-inflammatory molecules i.e. tumour necrosis factor, adiponectin and interleukins released by adipose tissue [33] as well as decreased nutrition to the intervertebral disc via atherosclerosis [34]. Indeed, fat mass or adipose tissue secret pro-inflammatory cytokines, such as tumor necrosis factor [35] that have been shown to be more prevalent in people with modic change [36] and muscle catabolism [37]. Likewise, in patients with chronic LBP, interleukins were present in abundance in those with mild disc degeneration diagnosed by MRI [38]. It is possible that changes in disc height, modic changes and muscle destruction play a distinctive role in the degeneration of tissue surrounding the spine and thus promote development of chronic pain conditions [13]. This is supported by the finding that C-reactive protein, a marker of chronic systemic inflammation, was associated with the odds of reporting LBP [39]. Moreover, atherosclerosis, largely driven by chronic inflammation [40], may decrease blood supply to the lumbar region, in the vertebrae and surrounding muscles [34]. These might result in reduced nutrition of the lumber intervertebral disc and result in tissue degeneration [34, 41]; which is in the pathway of LBP. For example, aortic atherosclerosis was shown to increase the risk for development of disc degeneration and was associated with the occurrence of LBP in participants of the Framingham cohort after 25-year follow-up [41]. Furthermore, central and peripheral pain sensitization also has a role in LBP [42, 43]. There is increasing evidence that systemic inflammation affects central and peripheral pain sensitization [44], which may be another mechanism for the adiposity-related meta-inflammatory processes [45]. Furthermore, it has been suggested that LBP could also cause less physical activity and thereby result in obesity, however in our analysis, to eliminate the effect of less physical activity, we have adjusted all our analyses.

The results of our study should be considered within the context of its limitations. We have used bio-impedance to measure body composition. Bio-impedance is fast, inexpensive, and does not require extensive operator training or cross-validation [46], however, bio-impedance depends on body hydration, which is difficult to assess and has a strong effect on the estimation of fat mass based on bioelectric impedance analysis [47]. As a result, any between-subject variability in hydration level in this study would have resulted in attenuation of the observed association between body composition, and LBP intensity and disability. Depression has been shown to predict the development of LBP and obesity [48], thus it is a confounding factor. Though we do not have data on depression as part of the AusDiab study, we have controlled our analysis for the mental component score of the SF-36, which can correctly identify 87% of cases of depression [49]. Furthermore, participants who responded to the Chronic Pain Grade Questionnaire had better health and were of lower socio-economic status compared with those who did not respond to the questionnaire, which is likely to have underestimated the association between measures of adiposity and LBP we observed. It is also possible that residual confounding may have accounted for the associations. However in this study, we were able to adjust for most of the well-known risk factors for LBP. Strengths of our study include the large sample size and wide age range of the cohort, and use of a validated measure of LBP intensity and disability. Furthermore, in this longitudinal study we have performed subgroup analysis leaving those experiencing moderate to very severe bodily pain at baseline and have observed the similar effect. This rules out the possibility of reverse causality that suffering from LBP at study induction may have been responsible for elevated adiposity measures.

## Conclusions

This study is the first large-scale, prospective, population-based cohort study demonstrating that fat mass is associated with LBP intensity and disability. This suggests both biomechanical and systemic factors associated with obesity contribute to the pathogenesis of LBP. Clarifying the mechanisms will be important for developing novel therapeutic approaches for the prevention and treatment of LBP.

## Additional files

Additional file 1: Table S1. General characteristics of participants who did not respond and who did respond to the low back pain questionnaire. **Table S2.** Correlation coefficients of baseline obesity and body composition measures with first follow-up obesity and body composition measures. (DOCX 19 kb)
Additional file 2: Chronic Pain Grade Questionnaire. (PDF 124 kb)

## Abbreviations

AusDiab: Australian Diabetes, Obesity and Lifestyle Study
BMI: Body mass index
CI: Confidence level
CPGQ: Chronic Pain Grade Questionnaire
HUNT: Nord-Trøndelag Health Study
LBP: Low back pain
MCS: Mental health component summary
OR: Odds ratio
PCS: Physical health component summary
SEIFA: Socio-Economic Indexes for Areas
SF-36: Short Form 36
