# Supplementary material for: Fat mass and fat distribution are associated with low back pain intensity and disability: results from a cohort study
Source: Arthritis Res Ther. 2017 Feb 10;19:26. doi: 10.1186/s13075-017-1242-z (PMC5301404; doi:10.1186/s13075-017-1242-z)
Supplement: Additional file 1: Table S1. — General characteristics of participants who did not respond and who did respond to the low back pain questionnaire. Table S2. Correlation coefficients of baseline obesity and body composition measures with first follow-up obesity and body composition measures. (DOCX 19 kb) [file 13075_2017_1242_MOESM1_ESM.docx]

**Table S1. General characteristics of participants who did not respond and who did respond to the low back pain questionnaire**

|  | **Not responded**  **n =** **1326** | **Responded**  **n =** **5058** | ***P*** |
| --- | --- | --- | --- |
| Age, years | 54.9 (14.5) | 49.2 (10.9) | <0.001 |
| Female, n (%) | 1020 (53.3) | 2479 (55.5) | 0.12 |
| University degree, n (%) | 462 (24.4) | 1630 (36.7) | <0.001 |
| Current smoker, n (%) | 238 (12.7) | 488 (11.1) | 0.02 |
| Socio-Economic Indexes for Areas (in lowest tertile %) | 743 (39.6) | 1324 (30.1) | <0.001 |
| Mental component score (SF 36) | 48.9 (9.9) | 49.3 (9.4) | 0.10 |
| BMI, kg/m^2^ | 27.5 (5.1) | 26.6 (4.7) | <0.001 |
| Waist circumference, cm | 92.8 (13.7) | 89.7 (13.6) | <0.001 |
| Percent of fat (%) | 33.5 (12.6) | 32.8 (11.8) | 0.08 |
| Fat mass, kg | 26.3 (13.1) | 25.6 (12.4) | 0.06 |
| Lean body mass, kg | 50.6 (12.2) | 50.8 (12.2) | 0.53 |

**Table S2. Correlation coefficients of baseline obesity and body composition measures with first follow-up obesity and body composition measures**

|  | **Correlation co-efficient**  **Baseline and first follow-up** | ***P* value** |
| --- | --- | --- |
| **Obesity measures** | | |
| Body mass index | 0.93 | <0.001 |
| Waist circumference | 0.89 | <0.001 |
| **Body composition measures** | | |
| Percentage fat | 0.95 | <0.001 |
| Fat mass | 0.90 | <0.001 |
| Fat-free mass | 0.97 | <0.001 |
